# Anterior Cerebral Artery and Middle Cerebral Artery Stroke in the Setting of Multiple Fusiform Aneurysms and Vertebrobasilar Dolichoectasia

**DOI:** 10.7759/cureus.83863

**Published:** 2025-05-10

**Authors:** Sarah K Radziewicz, Robbie D Buechler

**Affiliations:** 1 Neurology, Edward Via College of Osteopathic Medicine, Spartanburg, USA; 2 Neurology, Medical University of South Carolina, Lancaster, USA

**Keywords:** acute cerebrovascular accident, anterior cerebral artery (aca), cerebral fusiform aneurysm, middle cerebral artery (mca), radiology

## Abstract

We present a case of stroke in the setting of multiple fusiform aneurysms. This case is unique in several ways and highlights the risks, concerns, and treatment options of this lesser-known vascular disease. Fusiform aneurysms are an uncommon subtype of intracranial aneurysms compared to the traditional saccular type. Rarer is the prevalence of multiple fusiform aneurysms in one patient and in the anterior distribution. This case involves a 51-year-old man presenting with stroke-type symptoms and a moderate National Institutes of Health Stroke Scale score. Extensive radiographic testing demonstrated multifocal areas of restricted diffusion along the anterior cerebral and posterior middle cerebral artery distribution. Additionally, multiple fusiform aneurysms in the middle cerebral and anterior cerebral arteries were found, as well as a dolichoectasia in the vertebral basilar artery. This case will further elucidate the definition, diagnostic approach, and treatment modalities of fusiform aneurysms.

## Introduction

Intracranial aneurysms are one of the most common vascular defects of the brain [1]. They can be supratentorial or infratentorial and affect single or multiple arteries within one or both brain hemispheres [2]. They can also have different morphological classifications including saccular and fusiform [3]. Saccular aneurysms are common, while fusiform aneurysms only represent 3-13% of all intracranial aneurysms, with multiple aneurysms only seen in about 20% of all cases [2,3]. The majority of aneurysms are located in the vertebrobasilar system; when anterior, they are mainly in the middle cerebral artery (MCA) and internal carotid artery [3]. Fusiform aneurysms in the anterior circulation remain rare [3]. The most significant risk factors for the development of these aneurysms include atherosclerosis, arterial hypertension, infection, and smoking [2]. Connective tissue diseases that involve disorders of collagen and elastin metabolism can also contribute to the pathogenesis of fusiform aneurysms including Marfan and Ehlers-Danlos syndromes [3]. Many times, the word "dolichoectasia" is used interchangeably with "fusiform" aneurysm; however, by definition, these are different [2]. Dolichoectasia also encompasses the elongation of the artery in addition to its segmental widening, differentiating this from fusiform aneurysms [2]. Optimal management of unruptured intracranial fusiform arteries and dolichoectasia has been controversial due to the lack of randomized controlled trials that assess conservative management with endovascular and microsurgical techniques [4].

A well-defined but less frequent consequence of unruptured intracranial aneurysms is ischemic strokes [5]. Thrombus formation leading to transient ischemic attacks and ischemic strokes has been reported in giant, fusiform, and even small aneurysms [5]. The proposed mechanism of thrombus formation is due to the disruption of the internal elastic lamina which can advance to the adventitia, rupturing the aneurysm, or be contained by the media layer leading to an ischemic stroke [3]. With stroke remaining the second-leading cause of death in the world, research into any risk factor is pertinent [6]. The stroke burden, in terms of the absolute number of cases, has increased substantially over the past decade; however, less attention is given to those involving the anterior cerebral artery (ACA), which occurs in only <1.3% of all strokes [6,7]. ACA infarction can manifest as typical stroke symptoms including motor and sensory deficits [8]. Additionally, ACA strokes can have features of akinetic mutism, amnesia, and disconnection syndrome, differentiating this type of stroke from others [8]. Common risk factors for strokes include hypertension, hyperlipidemia, atrial fibrillation, and hypercoagulable states [9].

We present a case report of an acute cerebrovascular event in the setting of multiple fusiform aneurysms and vertebrobasilar dolichoectasia. This highlights the importance of a complete workup in stroke patients with fusiform aneurysms due to the coexisting comorbidities and increased risk for dissection or subsequent ischemic attack in the future.

## Case presentation

A 51-year-old right-handed African American man with a known history of atrial fibrillation, congestive heart failure, hyperlipidemia, hypertension, untreated sleep apnea, and previous myocardial infarction presented to the emergency department with right-sided hemiparesis and expressive aphasia with an onset of three days. Due to insurance and financial concerns, the patient was not able to afford anticoagulation in the setting of known atrial fibrillation, which was likely a contributing factor in the patient’s symptomatology.

Upon physical exam, blood pressure was elevated at 177/129 and rapid ventricular rate (RVR) atrial fibrillation was present on telemetry monitoring. Severe dense hemiparesis which was maximal in the lower extremity versus the upper extremity and expressive aphasia were present. The National Institutes of Health Stroke Scale score was 13, falling in the moderate stroke range. Due to the length of time of the symptoms he was deemed not a candidate for thrombolytic therapy; anticoagulation for his persistent atrial fibrillation with RVR was initially withheld due to the size of the stroke. Dual antiplatelet therapy was initiated instead. The patient was admitted and evaluated for stroke by neurology and cardiology. 

**Figure 1:**
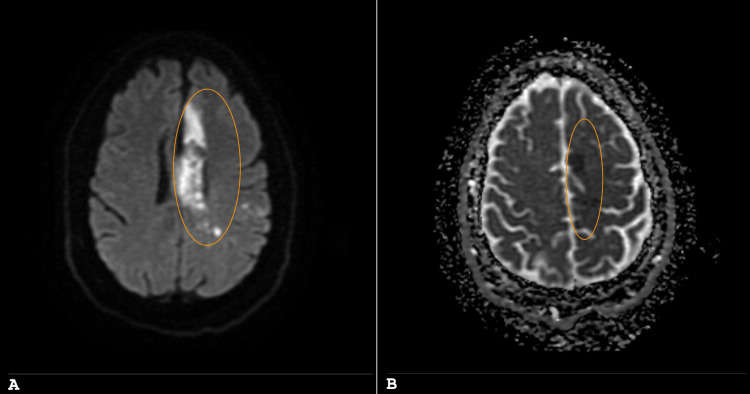
MRI of the brain. Panels A (diffusion-weighting imaging) and B (apparent diffusion coefficient mapping) demonstrate multifocal areas of restricted diffusion noted along the median left frontal cortex in the ACA distribution, as well as throughout the posterior left frontal and left parietal cortices (circled in orange). ACA: anterior cerebral artery.

**Figure 2:**
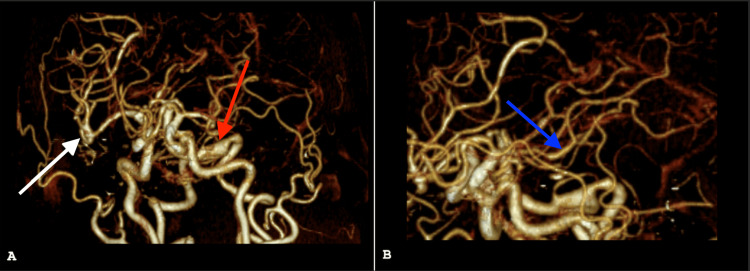
CT angiogram of head (coronal view with rotation). No evidence of proximal large vessel occlusion was seen, however imaging showed advanced atherosclerosis and severe stenosis of the A3 branch of the left anterior cerebral artery, with additional scattered areas of intracranial vascular stenosis. Panel A demonstrates dolichoectasia of the vertebrobasilar system (red arrow), and fusiform aneurysmal dilatation of the right MCA trifurcation (white arrow). Panel B demonstrates a fusiform aneurysmal dilatation of the A1 segment of the left ACA (blue arrow). MCA: middle cerebral artery, ACA: anterior cerebral artery.

The workup included an initial computed tomography (CT) scan, follow-up magnetic resonance imaging (MRI, Figures 1A, 1B), a magnetic resonance angiogram (not shown) with additional diffusion-weighted imaging, and two CT angiograms (Figures 2A, 2B).

This imaging demonstrated a large acute stroke in the anterior cerebral artery and middle cerebral artery distribution, fusiform aneurysms in the right MCA and left ACA, and dolichoectasia in the vertebral basilar system as shown in Figure 1 and Figure 2. These were without signs of dissection or bleeding, which was evaluated at various times with multiple methods to ensure this common risk factor associated with a fusiform aneurysm was not demonstrated, especially in the setting of atrial fibrillation that would require anticoagulation. Additionally, echocardiogram and telemetry demonstrated essentially normal ejection fraction and ongoing atrial fibrillation with RVR.

The patient was later discharged to rehabilitation. Upon discharge, he was started on anticoagulation for his atrial fibrillation and continuous positive airway pressure for his untreated sleep apnea. Additionally, interventional radiology and vascular surgery were consulted. Due to the recency of his stroke and the continued need for rehabilitation, they deemed him not a candidate for surgical intervention at this time and requested that he follow up after rehabilitation. The patient was also counseled on the purpose of anticoagulation therapy and the importance of medication compliance to prevent further issues in the future.

## Discussion

This case presentation aims to increase awareness of the stroke risk that exists when intracranial fusiform aneurysms are present. Fusiform intracranial aneurysms are circumferential arterial dilatations that are 1.5 times the normal diameter of an arterial segment, with any degree of tortuosity, without a definable neck [10]. These differ from the saccular type which has a balloon-like shape with an identifiable neck that separates the aneurysm from the host vessel [11]. Dolichoectasia are different from fusiform but are analogous due to their atherosclerotic and arterial hypertension etiology [2,12]. Fusiform intracranial aneurysms and vertebrobasilar dolichoectasia carry the risk of rupture and an additional risk of ischemia due to distal embolization or internal branch occlusion, as seen in this case discussion [11,13]. Treatment for fusiform aneurysms focuses on restoration of the arterial lumen, maintaining flow inside the aneurysm, and preventing occlusion of vessels that internally branch off [11]. The most effective treatment approach continues to be investigated, with previous systematic reviews finding no differences between morbidity of surgical and endovascular treatments for fusiform intracranial aneurysms [10]. In some studies, microsurgery has been postulated to yield better long-term angiographic results compared to endovascular procedures [10]. Presently, there are no universally effective treatments for vertebrobasilar dolichoectasia, but an endovascular flow diverter may be effective in select patients [14].

Special consideration should be taken for patients who present with these aneurysms in the setting of an ischemic stroke to investigate the presence of a possible dissection. Fusiform aneurysms and vertebrobasilar dolichoectasia are associated with significant morbidity and mortality [10,14]. Additional or repeat imaging, such as in this case, may be indicated to ensure a dissection is thoroughly evaluated. This case is further complicated by other stroke mechanisms, such as untreated sleep apnea, and limited access to care, including medical compliance with anticoagulation in the setting of atrial fibrillation. To help mitigate this issue, the patient and his family were provided with continued education on the reasoning for his anticoagulation therapy and the importance of following up with vascular surgery in the future.

## Conclusions

This case report highlights the rare presentation of an ACA and MCA ischemic stroke, in the setting of multiple fusiform aneurysms and vertebrobasilar dolichoectasia. It intends to educate on the special considerations that need to be evaluated when unruptured fusiform aneurysms are identified. Additionally, this case presents the multifactorial nature of ischemic stroke etiology. Limited access to care can impact medical compliance and have lasting impacts on the health of patients.
